# Reduced cortico-muscular beta coupling in Parkinson’s disease predicts motor impairment

**DOI:** 10.1093/braincomms/fcab179

**Published:** 2021-08-23

**Authors:** Nahid Zokaei, Andrew J Quinn, Michele T Hu, Masud Husain, Freek van Ede, Anna Christina Nobre

**Affiliations:** Oxford Centre for Human Brain Activity (OHBA), Wellcome Centre for Integrative Neuroimaging, Department of Psychiatry, University of Oxford, Oxford OX3 7JX, UK; Department of Experimental Psychology, University of Oxford, Oxford OX2 6GG, UK; Oxford Centre for Human Brain Activity (OHBA), Wellcome Centre for Integrative Neuroimaging, Department of Psychiatry, University of Oxford, Oxford OX3 7JX, UK; Department of Psychiatry, University of Oxford, Oxford OX3 7JX, UK; Department of Neurology, Nuffield Department of Clinical Neurosciences, University of Oxford, Oxford OX3 9DU, UK; Department of Experimental Psychology, University of Oxford, Oxford OX2 6GG, UK; Department of Neurology, Nuffield Department of Clinical Neurosciences, University of Oxford, Oxford OX3 9DU, UK; Oxford Centre for Human Brain Activity (OHBA), Wellcome Centre for Integrative Neuroimaging, Department of Psychiatry, University of Oxford, Oxford OX3 7JX, UK; Oxford Centre for Human Brain Activity (OHBA), Wellcome Centre for Integrative Neuroimaging, Department of Psychiatry, University of Oxford, Oxford OX3 7JX, UK; Department of Experimental Psychology, University of Oxford, Oxford OX2 6GG, UK

**Keywords:** Parkinson’s disease, cortico-muscular coherence, magnetoencephalography

## Abstract

Long-range communication through the motor system is thought to be facilitated by phase coupling between neural activity in the 15–30 Hz beta range. During periods of sustained muscle contraction (grip), such coupling is manifest between motor cortex and the contralateral forearm muscles—measured as the cortico-muscular coherence. We examined alterations in cortico-muscular coherence in individuals with Parkinson’s disease, while equating grip strength between individuals with Parkinson’s disease (off their medication) and healthy control participants. We show a marked reduction in beta cortico-muscular coherence in the Parkinson’s disease group, even though the grip strength was comparable between the two groups. Moreover, the reduced cortico-muscular coherence was related to motor symptoms, so that individuals with lower cortico-muscular coherence also displayed worse motor symptoms. These findings highlight the cortico-muscular coherence as a simple, effective and clinically relevant neural marker of Parkinson’s disease pathology, with the potential to aid monitoring of disease progression and the efficacy of novel treatments for Parkinson’s disease.

## Teaser

Zokaei et al. report reduced cortico-muscular coherence—coupling between cortex and muscle—in individuals with Parkinson’s disease compared to controls, during well-equated muscle contraction between the two groups. This reduction is related to motor symptoms in patients. This measure can provide a sensitive and objective measure of disease-related motor deficits.

## Graphical Abstract

**Graphical Abstract:**
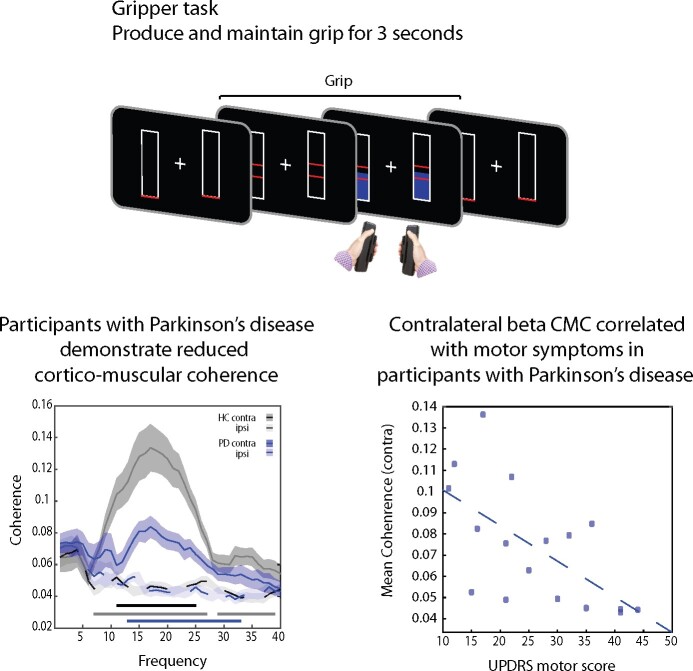

## Introduction

Parkinson’s disease is a neurodegenerative disorder characterized by a loss of dopaminergic neurons that alters neural activity in the basal ganglia, thalamus and the cortex.^1^ Excessive activity in the beta band (13–30 Hz) in the basal ganglia is a hallmark of Parkinson’s disease (see ref.^2^ for a review). Local field potential (LFP) recordings from subthalamic nucleus of individuals with Parkinson’s disease also show excessive beta activity, which is reduced following dopaminergic medication^3–6^ or deep brain stimulation (DBS) treatment.^7–9^

Contrary to the excessive beta activity in the basal ganglia, cortical beta in individuals with Parkinson’s disease may be attenuated instead.^10–13^ Moreover, prior studies have suggested that neural coupling between the cortex and the muscle—measured as the cortico-muscular coherence (CMC)—may also be reduced in Parkinson’s disease.^14–18^ The CMC quantifies the temporal relationship (phase coupling) between neural activity at a given frequency in the motor cortex and in the contralateral muscles, and is particularly pronounced during periods of steady muscle contraction.^19–22^ It is also impacted by other disorders involving motor impairments, such as stroke^23–25^ or amyotrophic lateral sclerosis,^26^^,^^27^ and may be used to track disease progression and therapeutic success.^14^^,^^18^^,^^28^^,^^29^

However, previous studies on changes in CMC in Parkinson’s disease have limitations. First, the required motor output during the period at which CMC was calculated has typically not been carefully equated between Parkinson’s disease and healthy control groups, making it difficult to disambiguate if differences in CMC were specific markers of disease or simply related to secondary differences in motor output. In addition, sample sizes were small in most prior studies. In many cases, measurements may also have been contaminated by concurrent DBS effects.

Here, we aimed to overcome these shortcomings by revisiting CMC in individuals with Parkinson’s disease in a task using carefully controlled and well-equated grip strength between a group of 17 individuals with Parkinson’s disease who were off medication and 17 matched healthy controls. We show that CMC is markedly reduced in individuals with Parkinson’s disease even when grip force was equated. Importantly, CMC strength also predicted motor impairments.

## Materials and methods

### Participants

The study was approved by the Oxford Research Ethics Committee as part of the National Research Ethics Service, and participants gave written informed consent to task procedures prior to participation.

Seventeen individuals with Parkinson’s disease and 17 age- and education-matched healthy control participants took part in this study (Table 1 for demographics). Participants in the Parkinson’s disease group were recruited from neurology clinics in Oxfordshire (UK). Exclusion criteria were being an active participant in an ongoing clinical drug trial, not tolerating coming off medication, taking psychotropic hypertensive or vasoactive medication or long-acting dopamine agonists, or a history of neurological or psychiatric disease other than Parkinson’s disease. Healthy control participants were recruited via the Friends of OxDare registry (https://oxfordhealthbrc.nihr.ac.uk/our-work/oxdare/public/become-a-friend/ Accessed 02 September 2021). All participants had normal or corrected-to-normal vision.

**Table 1:** Demographics, ACE III and UPDRS section III scores of Parkinson’s disease and healthy control participants

	Parkinson’s disease participants		Control participants		Δ
	Mean (SE)	Range	Mean (SE)	Range	*P*-value
Age (years)	66.5 (1.2)	52–78	69.7 (2.01)	54–80	n.s.
Education (years)	17.2 (1.5)	10–24	16.7 (0.9)	10–20	n.s.
ACE III	93.9 (4.03)	88–100	96 (3.3)	90–100	0.044
UPDRS section III	29.4 (3.23)	11–44	n/a	n/a	n/a
Daily levodopa equivalent dose	308 (135.7)	150–540	n/a	n/a	n/a

Participants with Parkinson’s disease were asked to withdraw from their dopaminergic medication at 7 p.m. the night before the experiment. The Addenbrooke’s Cognitive Examination (ACE III) test was administered as a general cognitive screening test to the Parkinson’s disease and healthy control groups (Table 1). Even though individuals with Parkinson’s disease scored significantly lower than healthy individuals, no participant had generalized cognitive impairment (as set by a cut-off point of 85). Unified Parkinson’s Disease Rating Scale (UPDRS) was administered prior to the scanning to all participants with Parkinson’s disease.

### Magnetoencephalography acquisition

Magnetoencephalography (MEG) data were acquired using an Elekta Neuromag system with 306 channels, at a sampling rate of 1000 Hz. Participants were seated comfortably in the MEG scanner and performed the task after being familiarized with the task procedure. Head position was tracked continuously using four fixed coil positions relative to the nasion and pre-auricular fiducial landmarks. The electrocardiogram was monitored by placing electrodes at both wrists. Saccades and blinks were monitored using recordings from electrodes around one of the eyes to derive the horizontal and vertical electro-oculogram, respectively. Muscle contraction was measured using bipolar electromyography (EMG) recordings at both forearms using electrodes placed ∼4 cm apart over the flexor digitorum superficialis of each arm, with reference electrodes on the lateral epicondyle (as in ref.^27^)

### Grip task and procedure

**Figure 1:**
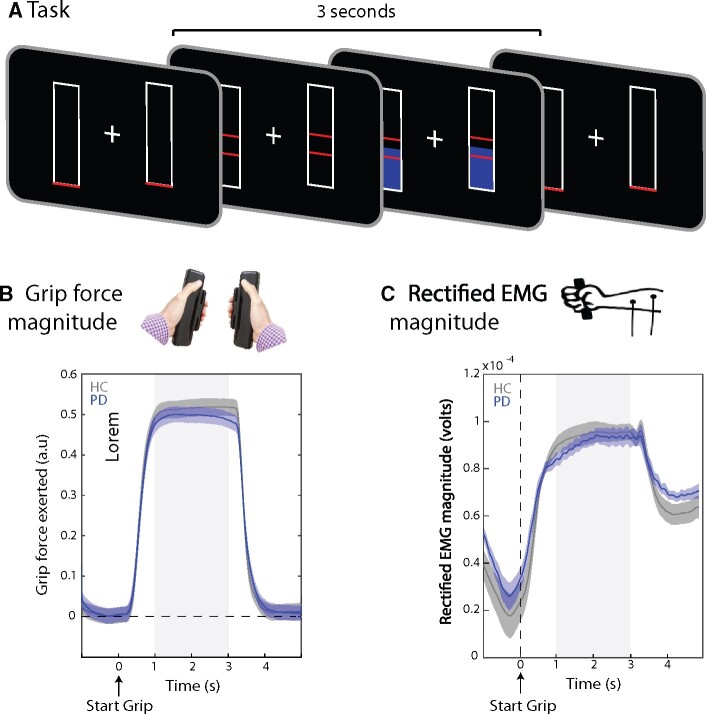
**Task schematic and exerted force.** (**A**) Schematic of the grip task in which participants had to exert bilateral auxotonic grip (isometric contraction) for a duration of 3 s. (**B and C**) The amount of force exerted was similar between healthy control (grey) and Parkinson’s disease (blue) participants, as measured in gripper output (**B**) and rectified EMG (**c**) (*n* = 17, *P* > 0.05). The shaded grey areas indicate the period of ‘stable grip’ used in the analysis of CMC.

Figure 1A contains a schematic of the grip task. In each trial, participants were presented with two bars on either side of the fixation cross corresponding to each hand (500 ms). The force to be exerted (target force) was then indicated by the height of two red lines on each bar (matched between the two hands). Across trials, participants had to produce and maintain a light grip of ∼12 Newtons or a strong grip of ∼17 Newtons. Using the same gripper devices, previous studies have reported no difference in maximum voluntary contraction in individuals with Parkinson’s disease and healthy individuals.^30^ Participants had to exert and maintain the grip for 3 s, after which time the red lines dropped to the bottom of both bars. Grip strength was recorded using MEG-compatible fibre-optic auxotonic force devices or ‘grippers’ (Current Designs, USA). Participants received direct visual feedback on the force they exerted on each gripper (Fig. 1a, blue bars). Participants could relax for 2000 ms between successive grip trials. All participants performed 120 trials of the task (60 per force condition) across 12 blocks of 10 trials each.

Some participants across both Parkinson’s disease and Healthy control (HC) groups had difficulty reaching and/or maintaining the required stronger grip force in many trials. We therefore restricted our analyses to the light-grip trials, which all participants could comfortably manage. Both groups successfully completed the task in time, and none of the participants reported being fatigued by task procedure.

Trials in which participants clearly did not reach or maintain the required grip level (in which the force exerted reached zero during the grip duration, after baseline correction) were excluded from analysis. Both Parkinson’s disease and HC participants could perform the task successfully, with an average of 56 (out of 60) trials completed by HC participants (SD: 3.12) and 56.7 trials by Parkinson’s disease participants (SD: 4.2). There was no significant difference between the number of usable trials completed by HC and Parkinson’s disease participants [*t*(32) = 0.61, *P* = 0.5].

### Pre-processing

Spatial signal space separation and movement compensation were applied using Neuromag’s Maxfilter software to separate signals arising from inside versus outside of the MEG helmet (minimizing extracranial noise) and compensate for the effects of head movements using continuous head position measurements. Data were then converted to fieldtrip format, checked manually to ensure no problems arose during the Maxfilter pre-processing stage, and downsampled to 250 Hz. Low frequency drift was removed by using a 0.1-Hz high-pass filter. The downsampled data were epoched; and Independent Component Analysis was used to remove artefacts associated with blinks, saccades and heartbeat. Any remaining artefacts were rejected following visual inspection of the data. To investigate muscle contraction during the grip period, we additionally constructed a channel with high-pass filtered (40 Hz cut-off) and baseline-corrected EMG traces (averaged between the left and right arm).

### CMC estimation

The CMC is a measure of phase coupling (normalized to range from 0 to 1) between the cortical motor MEG signal and the corresponding contralateral muscular EMG signal that is particularly prevalent during continuous, isometric and contraction.^19^^,^^21^^,^^22^^,^^27^^,^^31^ We used the rectified raw EMG traces when calculating CMC. CMC was calculated using the FieldTrip toolbox,^32^ using the data from the steady contraction window between 1000 and 3000 ms after the onset of the grip instruction. CMC was calculated between 1 and 40 Hz, in 0.25 Hz steps. A multi-taper method^33^ was applied to achieve ±5 Hz spectral smoothing. We focused our analysis of CMC magnitude on MEG signals from the 12 combined planar-gradiometer channels (6 per hemisphere) which a previous, independent, study from our lab showed to be particularly sensitive to motor cortical activity (4). We used the same set of left and right channels for both Parkinson’s disease and HC groups.

### Statistical analysis

To avoid issues with multiple comparisons, we contrasted the CMC spectra between groups using a cluster-based permutation analyses,^34^ with 5000 permutations.

### Data availability

The conditions of our ethics approval do not permit public archiving of the data supporting this study, and sharing data require a formal data-sharing agreement in accordance with ethics procedures governing the re-use of sensitive data. Readers seeking access to the data should contact the first author.

## Results

We first ascertained that both the HC and the Parkinson’s disease group had similar performance on the grip task (Fig. 1A). As shown in Fig. 1B, participants demonstrated a clear modulation of grip strength during the instructed grip period. Importantly, both groups achieved similar levels of grip force. We found no signification differences in mean grip force (Fig. 1B) or in muscle contraction as measured through rectified EMG signals (Fig. 1C) during the time of the grip (no significant clusters).

**Figure 2:**
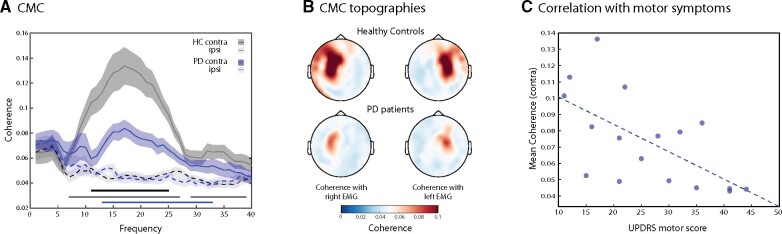
**Cortico-muscular coherence (CMC).** (**A**) CMC between the forearm EMG and contralateral (solid lines) and ipsilateral (dashed lines) motor cortex in the 1–3 s stable grip period (shaded areas in Fig. 1B and C). Contralateral beta CMC was significantly reduced in individuals with Parkinson’s disease (blue line—*n* = 17, significant cluster: 13–33 Hz, cluster *P* < 0.0001) compared to healthy control participants (grey line—*n* = 17, significant cluster: 7–27, 29–39 Hz, cluster *P* < 0.0001), using cluster-based permutation analysis (significant cluster: 11–25 HZ, cluster *P* = 0.008). Contralateral CMC was also significantly different between groups (black line, significant cluster: 11–25 HZ, cluster *P* = 0.008). (**B**) Topographical distributions of CMC in both participant groups, separately for coupling with the left and right EMG. (**C**) Mean contralateral beta CMC, averaged over the significant cluster in a 11–25 Hz was significantly correlated with motor symptoms in the Parkinson’s disease group, as measured by the UPDRS (*n* = 17, *r* = −0.63, *P* = 0.007).

We next turned to our primary outcome measure of interest: cortico-muscular phase coupling, CMC. In line with prior studies of CMC,^19^^,^^21^^,^^22^^,^^27^^,^^31^ we observed particularly pronounced coupling between sensors over motor cortex and the contralateral muscle in the beta band. Frequency profiles were similar between groups. Taking the coupling with the ipsilateral muscle as our neutral ‘baseline’, we found significant contralateral (versus ipsilateral) coupling in both Parkinson’s disease (13–33 Hz, cluster *P *< 0.0001; Fig. 2a, blue line) and HC (7–27 Hz, 29–39 Hz, cluster *P *< 0.0001; Fig. 2A, grey line) participants. Critically, however, despite similar grip strength (Fig. 1B), individuals with Parkinson’s disease demonstrated a marked reduction in beta coupling between the cortex and contralateral muscle, as confirmed by a significant group difference (cluster-based permutation significant cluster: 11–25 Hz, cluster *P *=* *0.008; Fig. 2a, black line). Moreover, there was no difference in CMC in the affected versus the unaffected side in individuals with Parkinson’s disease (Supplementary Fig. 1, in 15 individuals with unilateral symptom profile).

The reduction of CMC between cortex and contralateral muscle was also evident when considering its topographical distribution (Fig. 2B). In both groups, coherence was localized to the same contralateral motor channels, but was considerably weaker in Parkinson’s disease compared to HC participants.

We tested whether the reduction in contralateral beta coupling in Parkinson’s disease compared to HC participants might be a relevant clinical marker. Within the Parkinson’s disease group, the magnitude of CMC was negatively correlated to motor symptoms as measured by the UPDRS section III (motor symptoms) (Fig. 2C). Lower (‘more reduced’) coupling was associated with more severe symptoms (*r* = −0.63, *P* = 0.007).

In addition to robust group differences in cortico-muscular coupling, there were also significant group differences in cortical power (Supplementary Fig. 2a; with one cluster between 6 and 8 Hz, cluster *P *=* *0.05, and another between 15.5 and 37 Hz, cluster *P *=* *0.0019), as well as increased temporal ‘variability’ in the rectified EMG (likely a reflection of tremor Supplementary Fig. 2b). Variability was measured by calculating variance in the rectified EMG, across 200 ms window that we advanced over the data in steps of 50 ms. Critically, however, these variables did not predict CMC or UPDRS section III scores (Supplementary Fig. 3). Moreover, when taking these variables into account, by partialling out mean power and EMG variability, the correlation between CMC and motor symptoms of UPDRS section III scores remained significant (*r *=* *−0.61, *P *=* *0.015). Finally, there was no significant relationship between CMC and years of diagnosis (*r *=* *0.15, *P *=* *0.59) or daily levodopa equivalent dose (*r *=* *−0.12, *P *=* *0.68).

## Discussion

The results of this study demonstrated a marked reduction in beta CMC during a period of controlled sustained grip in the Parkinson’s disease group, despite comparable grip magnitude to that of a matched healthy control group. Moreover, the reduced CMC was related to motor symptoms in the Parkinson’s disease group as measured by the UPDRS section III, so that individuals with lower CMC in the beta range also displayed worse motor symptoms.

Remarkably, the reduced CMC in individuals with Parkinson’s disease did not impact their ability to perform the grip task in any obvious way. Even though the precise functional role of CMC is not fully understood,^35^ it has been shown to relate to both the quality and precision of motor performance as well as to skill learning in healthy participants.^36–38^ Yet, the current work presents a curious situation in which a clear reduction in CMC occurred despite largely preserved grip performance. Importantly, the low magnitude of the required grip in our task was sufficient to reveal robust group differences in CMC while minimizing changes to overall motor performance. This advantageous context enabled us to re-evaluate changes in CMC in Parkinson’s disease versus HC, while minimizing the contribution of any group differences attributable to secondary differences in motor behaviour.

Deciphering the exact neurological mechanisms that underlie the observed group differences—and their link to disease severity—remains an important target for future studies. Previous studies have suggested that reduced CMC in Parkinson’s disease may arise from deficits in programming of movement that is reflected in the loss of synchronized oscillatory activity in muscle discharge.^39^^,^^40^ More specifically, it has been hypothesized that CMC loads on the same pathways that are relevant and affected in bradykinesia.^41^ In addition, proprioceptive processing has been proposed to contribute to CMC^40^ and may also contribute to the observed differences.

Motor output (such as tremor present in participants with Parkinson’s disease) also provides continuous motor/sensory input and could thereby reduce beta oscillations and as consequence result in a reduction in CMC. This intriguing possibility warrants further testing. However, attenuation of CMC by input associated with tremor was unlikely to account for our findings. In individuals with lateralized tremor in the Parkinson’s disease group, the effects were equivalent when analysing CMC in relation to the hand affected and unaffected by tremor (Supplementary Fig. 1). Moreover, when we regressed out participant-specific EMG variability—as a proxy for tremor—during the gripping period, we found that the relationship between clinical symptoms and CMC persisted.

Our findings build on related previous studies,^14–18^ while also controlling for important prior limitations. First, we measured CMC during a period of steady muscle contraction that was comparable between Parkinson’s disease and HC participants. In most prior studies, participants were usually asked simply to extend their wrist or to contract their forearm, with no objective measure on the strength of muscle contraction. Moreover, sample sizes were typically smaller, and in some cases artefacts associated with concurrent DBS may have been included.^14^^,^^16^^,^^18^

After controlling for these shortcomings, our results revealed a possible relationship between the magnitude of CMC and the clinical symptoms in Parkinson’s disease. The CMC proved to be a particularly sensitive measure. Interestingly, there was no relationship between clinical symptoms of Parkinson’s disease and mean power in the beta range, despite a clear reduction of beta power in Parkinson’s disease compared to control participants.^10^^,^^12^^,^^13^ Furthermore, the relationship between CMC and motor impairment in participants remained even after controlling for changes in beta power. Thus, it is possible that our task and the CMC, by loading on relevant pathways,^19^^,^^20^ provide a more sensitive and objective measure of Parkinson’s disease-related motor deficits, compared to beta power.^27^ The simple and well-controlled gripper task employed here, in conjunction with MEG (or EEG) and EMG recordings, may therefore provide a convenient and effective set-up in which to obtain a sensitive CMC marker to monitor disease progression or when testing the influence of novel treatments for Parkinson’s disease. It will be interesting to investigate whether changes in cortico-muscular coupling are already present in early stages of the disease or in individuals at risk of developing Parkinson’s disease (such as individuals with Rapid Eye-movement Sleep behavioural Disorder), thereby possibly providing a valuable early marker aiding risk assessment, stratification, diagnosis and disease prognosis.

## Supplementary material

Supplementary material is available at *Brain Communications* online.

## Funding

This research was funded in whole, or in part, by the Wellcome Trust (104571/Z/14/Z to A.C.N. and 098282/Z/12/Z to M.H.), and the British Academy (N.Z.), and supported by the National Institute for Health Research (NIHR) based at Oxford University Hospitals NHS Trust and the NIHR Oxford Health Biomedical Research Centre. The Wellcome Centre for Integrative Neuroimaging is supported by core funding from the Wellcome Trust (203130/Z/16/Z). For the purpose of open access, the author has applied a CC BY public copyright licence to any Author Accepted Manuscript version arising from this submission.

## Competing interests

The authors report no competing interests.

## Supplementary Material

fcab179_Supplementary_Data

## Abbreviations

ACE III =: Addenbrooke’s Cognitive Examination III
CMC =: cortico-muscular coherence
DBS =: deep brain stimulation
MEG =: magnetoencephalography
UPDRS =: Unified Parkinson’s Disease Rating Scale
