# Supplementary material for: Reduced cortico-muscular beta coupling in Parkinson’s disease predicts motor impairment
Source: Brain Commun. 2021 Aug 23;3(3):fcab179. doi: 10.1093/braincomms/fcab179 (PMC8421699; doi:10.1093/braincomms/fcab179)
Supplement: fcab179_Supplementary_Data [file fcab179_supplementary_data.pdf]

Supplementary Material (Figures S1-S3)

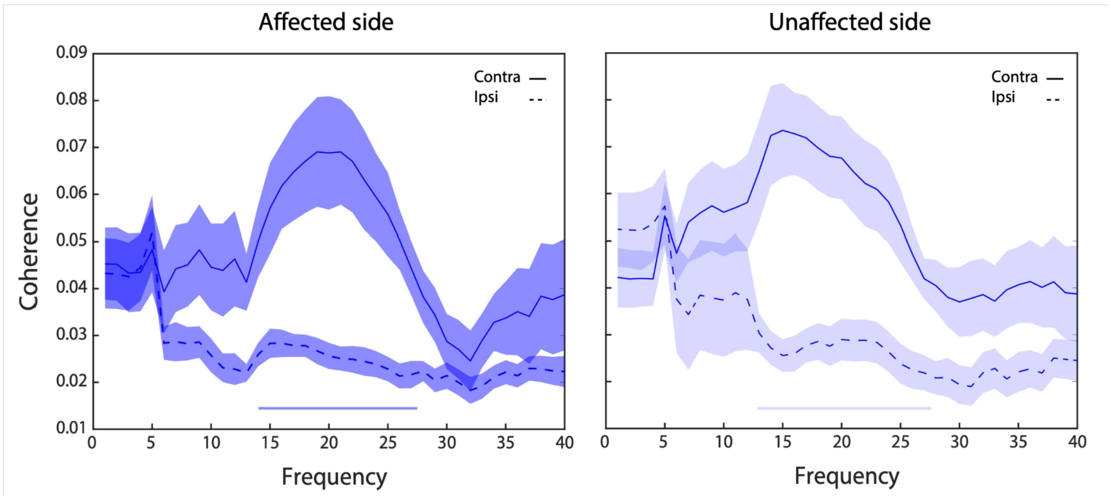

Supplementary Figure 1. Cortico-muscular coherence (CMC) between the forearm EMG and contralateral (solid lines) and ipsilateral (dashed lines) motor cortex to the affected (darker blue – left graph) and unaffected (lighter blue – right graph) sides during the 1-3 s stable-grip period (shaded areas in Fig. 1b-c) in fifteen participants with Parkinson's disease with tremor present in one hand as measured by questions 3.15, 2.16 and 3.17 in the UPDR III. Contralateral beta CMC was significantly higher than ipsilateral beta CMC for both affected and unaffected sides. There was no significant difference in contralateral CMC for the affected and unaffected sides.

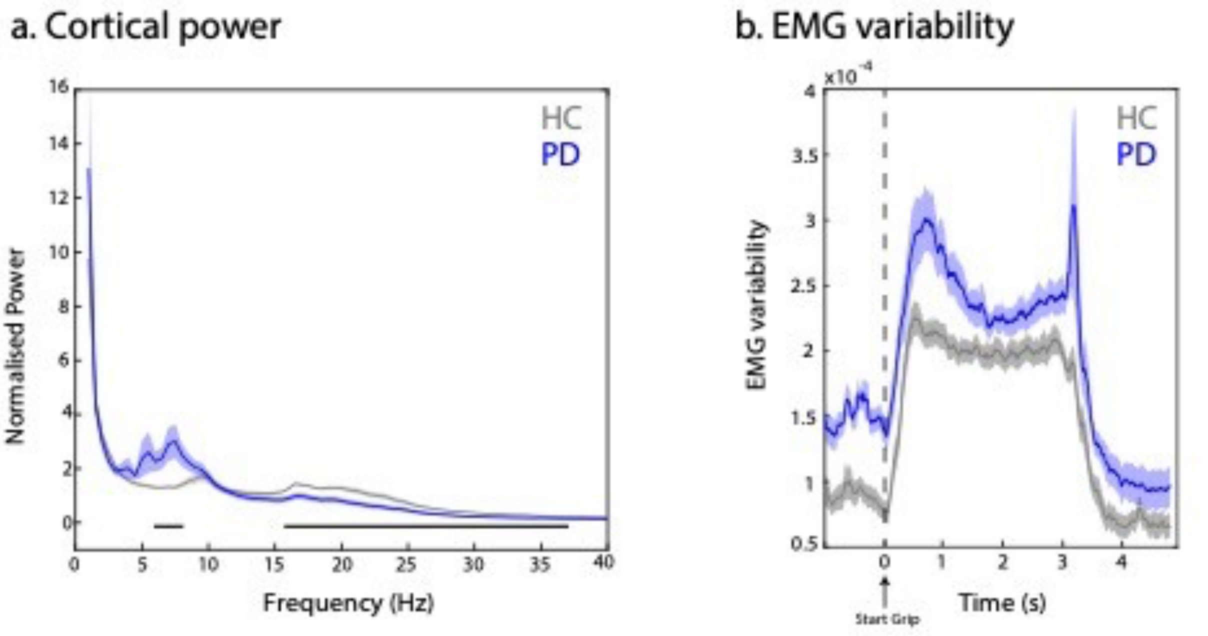

Supplementary Figure 2. a) Normalised power (power at a given frequency divided by the average power at all other frequencies) in the pre-defined motor channels during the task. In line with previous studies, participants with PD (blue) had significantly stronger alpha power (6-8 Hz, cluster *p*= 0.05) and reduced beta power (15.5-37 Hz, cluster *p*= 0.0019). Mean beta power in PD participants was used as a covariate for any analysis examining the relationship between CMC and disease-related measures (see also **Figure S3**). It is important to note that even though changes to the alpha cluster in the PD group here may reflect tremor in these individuals (considering that only motor channels were used to obtain cortical power), similar changes in alpha power occur in non-motor channels in PD (Stoffers et al., 2007) and have also been reported in other neurogenerative conditions, such as Alzheimer's disease (e.g. Osipova et al., 2004; Poza et al., 2004; Hughes et al., 2019). b) Participants with PD (blue) had significantly more variable EMG compared to healthy controls, though this was not restricted to the grip period. This variability may reflect tremor in some of these individuals. EMG variability at the time of the grip was used as a covariate for any analysis examining the relationship between CMC and disease-related measures (see also **Figure S3**).

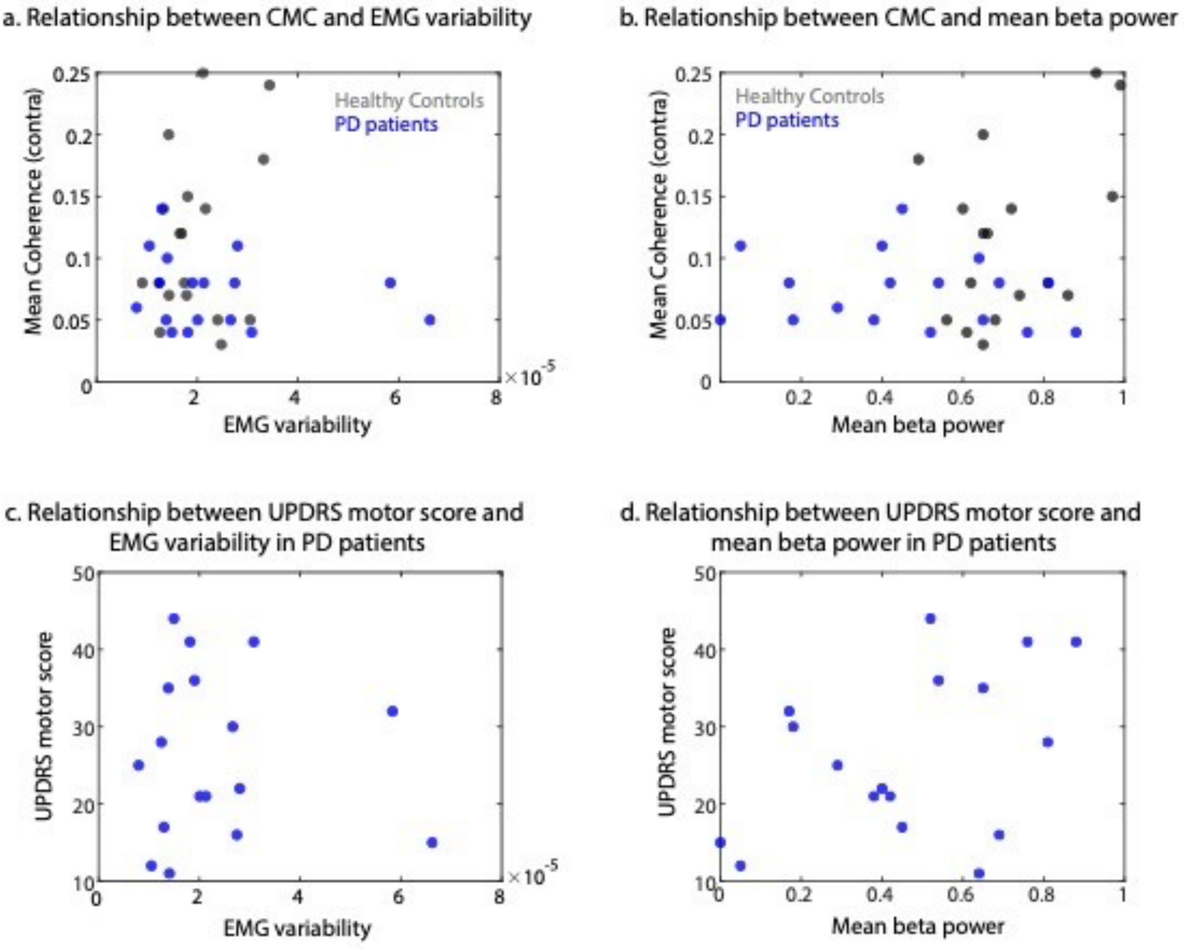

Supplementary Figure 3. There were no relationships between CMC and EMG variability (both groups r= -0.010, *p*= 0.96, PD group only: r= -0.19, *p*= 0.46) (a), or between CMC and cortical beta power (r= 0.32, *p*= 0.063) (b), across participants. In participants with PD, motor symptoms as measured by the UPDRS section III (motor symptoms), also did not correlate with EMG variability (r= -0.045, *p*= 863) (c) or with mean cortical beta power (r=0.43, *p*= 0.086) (d).

Supplementary references

M. W., Nobre, A. C., Rowe, J. B., Maestú, F., & the BioFIND Working Group. (2019). Biomagnetic biomarkers for dementia: A pilot multicentre study with a recommended methodological framework for magnetoencephalography. *Alzheimer's & Dementia: Diagnosis, Assessment & Disease Monitoring*, *11*(1), 450–462. https://doi.org/10.1016/j.dadm.2019.04.009

Marsden, J., Limousin-Dowsey, P., Fraix, V., Pollak, P., Odin, P., & Brown, P. (2001). Intermuscular coherence in Parkinson's disease: Effects of subthalamic nucleus stimulation. *Neuroreport*, *12*(6), 1113–1117. https://doi.org/10.1097/00001756-200105080-00013

Osipova, D., Ahveninen, J., Jensen, O., Ylikoski, A., & Pekkonen, E. (2005). Altered generation of spontaneous oscillations in Alzheimer's disease. *NeuroImage*, *27*(4), 835–841. https://doi.org/10.1016/j.neuroimage.2005.05.011

Poza, J., Hornero, R., Abásolo, D., Fernández, A., & Mayo, A. (2008). Evaluation of spectral ratio measures from spontaneous MEG recordings in patients with Alzheimer's disease. *Computer Methods and Programs in Biomedicine*, *90*(2), 137–147. https://doi.org/10.1016/j.cmpb.2007.12.004
